# Hepatocellular Adenoma With Hepatocyte Nuclear Factor-1 Alpha (HNF-1α) Mutation: A Case Report

**DOI:** 10.7759/cureus.64017

**Published:** 2024-07-07

**Authors:** Pratiksha Moliya, Hasan Al-Obaidi, Hussein Harb, Utsav Moliya, Mehta Asit

**Affiliations:** 1 Internal Medicine, Jamaica Hospital Medical Center, New York, USA; 2 Basic Sciences, Ross University School of Medicine, Bridgetown, BRB; 3 Internal Medicine, College of Medical Sciences, Bharatpur, NPL; 4 Gastroenterology, Jamaica Hospital Medical Center, New York, USA

**Keywords:** benign liver tumours, multidisciplinary management, treatment strategies, magnetic resonance imaging (mri), hepatocellular adenoma (hca)

## Abstract

Hepatocellular adenoma (HCA) is an uncommon benign liver tumor that exhibits a variety of subtypes, each distinguished by unique molecular alterations. This case report describes a 43-year-old man with a history of alcoholism who presented with stomach pain. Imaging revealed multiple hepatic lesions and sigmoid colon inflammation, while laboratory tests showed mild neutrophilic leukocytosis and elevated liver enzymes. Tumor markers were normal. A liver biopsy confirmed HCA with hepatocyte nuclear factor-1 alpha (HNF-1α) inactivation, characterized by negative immunostaining for glutamine synthetase, nuclear beta-catenin, serum amyloid A, C-reactive protein, and liver fatty acid-binding protein (L-FABP). This case is unique due to the patient's gender and the absence of typical risk factors such as abnormal hormone levels. HCAs in males, particularly with HNF-1α inactivation, are rare and pose diagnostic challenges. Comprehensive diagnostic approaches, including biopsy and immunohistochemical analysis, are crucial for accurate subtype identification. The potential for malignant transformation, particularly in male patients, underscores the need for vigilant monitoring and appropriate management. This case highlights the importance of considering HCA in differential diagnoses regardless of gender and typical risk factors, contributing valuable insights into the diverse presentations and risks associated with HCA, and emphasizing the need for awareness and further research to improve diagnosis and management of this rare condition.

## Introduction

Hepatocellular adenoma (HCA) is a rare benign liver tumor that primarily affects women of reproductive age who use oral contraceptives. Since 2002, HCAs have been recognized as a diverse set of benign neoplastic hepatocellular proliferations comprising several subtypes [1]. Based on morphological and immunophenotypical traits, HCAs are categorized into five main subtypes: inflammatory HCA, β-catenin mutant HCA, sonic hedgehog HCA, hepatocyte nuclear factor-1 alpha (HNF-1α) inactivated HCA (H-HCA), and unclassified HCA. These subtypes are characterized by specific pathways, including the HNF-1α pathway, β-catenin signaling, and the IL-6/JAK/STAT pathway [2]. A newly discovered subtype called Sonic HCA (shHCA) has also been documented, though further clarification is needed for its classification [3].

The two most common complications of HCAs are hemorrhage and malignant transformation. The risk of malignant transformation into hepatocellular carcinoma (HCC) increases with male gender, larger HCA tumor size, and specific HCA subtypes, particularly those with β-catenin mutations, which carry a risk as high as 40% [1]. Differentiating HCA from well-differentiated HCC can be challenging, potentially leading to misdiagnosis as "atypical/borderline HCA" [1]. Recent research by Sasaki et al. revealed that cases of alcoholic cirrhosis with associated hepatic nodules expressed immunohistochemistry serum amyloid A, linked to the inflammatory subtype of HCA, and some cases expressed signal transducer and activator of transcription 3 (STAT3) mutations, implicated in HCA pathogenesis [4]. This suggests a unique subtype of inflammatory hepatocellular tumors may emerge in the context of alcohol-induced cirrhosis, requiring further research to prevent misdiagnosis with inflammatory HCA subtypes [2].

This case report presents a unique instance of HCA in a male with a distant history of alcohol abuse, no abnormal lab findings, and atypical imaging findings not indicative of H-HCA. The rarity of this case, combined with its atypical presentation and limited associated risk factors, provides valuable insights into the literature.

## Case presentation

A 43-year-old man reported having stomach pain for a week. He disclosed no noteworthy medical or familial history but did admit to a history of alcoholism, marijuana use, and cigarette smoking. He was hemodynamically stable when he arrived at the hospital, and the emergency department gave him painkillers and intravenous hydration.

**Figure 1:**
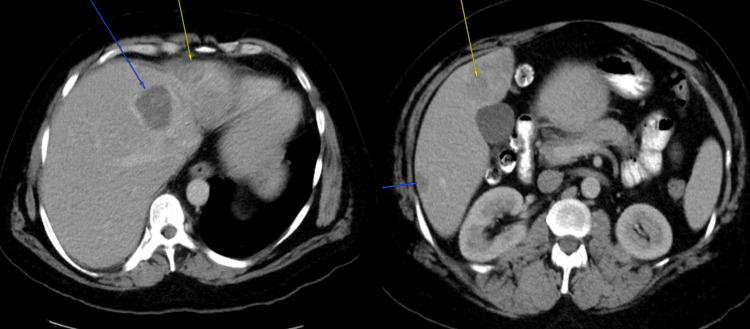
Numerous lesions, indicated by blue and yellow arrows, measuring between 2.2 and 6.4 cm, were observed in various parts of the liver during an abdominal computed tomography (CT) scan.

Numerous lesions measuring between 2.2 and 6.4 cm were seen in various parts of his liver during an abdominal computed tomography (CT) scan (Figure 1). Concern for an underlying mass was also raised by the observation of a mural lesion and surrounding inflammation in the sigmoid colon. A modest case of neutrophilic leukocytosis with a white blood cell count of 11.3K was observed in the laboratory. Additionally, testing for liver function showed increased levels of alkaline phosphatase (ALP) at 259 IU/L and alanine transaminase (ALT) at 76 IU/L. The patient was released from the emergency department with a planned outpatient appointment with a gastroenterologist. More testing was necessary because there were several liver abnormalities, which raised the possibility of liver metastasis. To rule out the main lesions, colonoscopy and gastroscopy were done, but the outcomes were not particularly noteworthy.

Following tumor marker evaluations, the levels of alpha-fetoprotein (AFP), carbohydrate antigen 19-9 (CA 19-9), and carcinoembryonic antigen (CEA) recovered to normal. However, the patient was referred to the interventional radiology department for a liver biopsy because of the hepatic lesions' persistence. HCA was found in the liver biopsy results, and tests for glutamine synthetase, nuclear beta-catenin, serum amyloid A, C-reactive protein, and liver fatty acid-binding protein (L-FABP) all showed negative immune-staining results. These results were in line with a HCA including a mutation in HNF-1α.

Additionally, the diagnosis was confirmed by immune staining for CD34, which revealed patchy capillarization of sinusoids inside the lesion. Crucially, there was no serological proof of an active case of hepatitis B or C. HCA was conclusively diagnosed as a result of the thorough evaluation, which gave important new information about the patient's health and directed future treatment choices. The patient was discharged with full resolution of his symptoms, no complaints, and questions answered. He was referred to a hepatologist but was lost to follow-up.

## Discussion

HCA is a benign liver tumor characterized by the monoclonal proliferation of hepatocytes. The etiology of HCAs involves hormonal influences and genetic predispositions, with significant risk factors including oral contraceptive use and anabolic androgenic steroids [5]. Other factors associated with HCAs include chronic alcoholism, metabolic syndrome, hemochromatosis, obesity, and familial adenomatous polyposis [6,7]. Genetic mutations have led to the identification of various HCA subtypes, with inflammatory HCA being the most prevalent, followed by HNF-1α inactivated HCA (H-HCA), β-catenin mutated HCA, and sonic hedgehog HCA [2,3].

Our case is unique not only due to the patient's gender but also the absence of abnormal androgen and estrogen levels, despite a history of alcoholism. HCAs are more common in Western countries, with an incidence of 3.4 per 100,000 persons in North America and Europe, compared to lower frequencies in Asia [8]. The European Association for the Study of the Liver (EASL) reports a significant female predominance, with a female-to-male ratio of approximately 10:1 [9].

Clinically, HCAs are often asymptomatic and discovered incidentally on imaging [5]. Large adenomas may cause right upper quadrant abdominal pain and can rupture, leading to bleeding and requiring emergency surgery [10]. H-HCA, the subtype identified in this case, is characterized by inactivation of the HNF1α transcription factor, leading to metabolic alterations and a homogenous steatotic phenotype without inflammatory infiltrates. The presence of multiple H-HCA lesions is referred to as liver adenomatosis.

Magnetic resonance imaging (MRI) is the most effective technique for diagnosing HCAs and differentiating subtypes based on pathologic features. In our case, a CT scan revealed multiple liver lesions and inflammation in the sigmoid colon, prompting a biopsy to confirm H-HCA. The absence of typical steatosis on imaging and the presence of surrounding inflammation indicated a need for comprehensive investigation [11].

The treatment of HCAs is guided by the Bordeaux classification, which categorizes HCAs based on genetic and phenotypic characteristics [12-13]. The American College of Gastroenterology recommends resection for HCAs ≥5 cm due to the risk of hemorrhage and malignant transformation, while smaller HCAs may be managed conservatively. β-catenin-activated HCAs, regardless of size, should be referred early for resection due to a higher malignancy risk [14]. In cases where surgery poses a high risk, alternative interventions like embolization may be considered [15].

The prognosis depends on the HCA subtype, size, and patient gender. H-HCAs may regress or decrease in size with the discontinuation of hormonal medications, but complete regression is unlikely, with up to 45% of patients experiencing hemorrhage, which has a mortality rate of up to 10% [5]. Larger adenomas (>5 cm) have a higher risk of hemorrhage and malignant transformation [13]. Male patients with HCAs have a higher risk of malignant transformation, with studies suggesting a significantly high 10-year cumulative risk of progression to HCC, particularly in men older than 50 years at diagnosis [16]. Therefore, it is crucial to thoroughly evaluate male patients with suspected or confirmed HCA due to the increased malignancy risk.

In conclusion, this case of HCA in a male patient with a history of alcoholism and an atypical presentation highlights the importance of thorough evaluation and the need for further research to better understand and manage such unique presentations.

## Conclusions

This case report highlights the unique presentation of HCA in a male patient with a history of alcoholism and atypical clinical features. The absence of typical risk factors such as abnormal androgen and estrogen levels, combined with the rarity of HCA in males, underscores the importance of considering HCA in differential diagnoses regardless of gender. The patient's presentation with multiple hepatic lesions and surrounding inflammation further emphasizes the need for comprehensive diagnostic approaches, including biopsy and detailed immunohistochemical analysis, to accurately identify HCA subtypes. Given the potential for malignant transformation, particularly in male patients, vigilant monitoring and appropriate management strategies are crucial. This case contributes valuable insights to the literature, highlighting the need for awareness and further research into the diverse presentations and risks associated with HCA.
